# The Social Housing Crisis and the Barriers to Developing Dementia-Friendly Communities in Chile

**DOI:** 10.3389/fpubh.2021.662364

**Published:** 2021-08-24

**Authors:** Daniel A. Jiménez, Francisca Cancino-Contreras

**Affiliations:** ^1^Department of Neurological Sciences, Faculty of Medicine, University of Chile, Santiago, Chile; ^2^Department of Neuroscience, Faculty of Medicine, University of Chile, Santiago, Chile; ^3^Servicio de Neurología, Hospital Salvador, Santiago, Chile; ^4^Francisca Cancino-Contreras, Facultad de Arquitectura, Diseño y Construcción, Universidad de las Américas, Providencia, Chile

**Keywords:** dementia-friendly communities, Chile, social housing, dementia, neighbourhood

## Abstract

Interaction with living place and neighbourhood is one of the cornerstones for creating dementia-friendly communities (DFC). Chile has one of the largest proportions of older adults in Latin America and is currently facing an increase in the number of people with dementia. In this context, the Chilean government has launched a national strategy that involves actions in the health and social care system, including the promotion of DFC. From a multisectoral approach, social and environmental aspects involving engagement with local communities and access to social connections and services are directly related to urban policies. This perspective article focuses on urban aspects of social housing policy, such as placement, networks, affordability and the relationship between subsidy structure and adequate housing provision in a country with a qualitative housing deficit of around 1,200,000 units and where a large proportion of people with dementia and their families live in poverty. We identified several barriers to delivering appropriate environments for people living with dementia in relation to a two-fold problem: (a) the social housing subsidy displaces caregivers and/or older adults to satellite towns where social connections and access to services and urban equipment are lost; and (b) people resisting displacement live in overcrowded neighbourhoods where dementia is a common problem. In both scenarios, a detrimental environment and social conditions directly affect the quality of life of elderly people living with dementia and their caregivers.

## Introduction

Chile has one of the largest proportions of older adults (over 60 years of age) in Latin America and is currently facing an increase in the prevalence of dementia and other non-communicable chronic diseases (1). The rapid ageing of Chile's population has been driven by an increase in life expectancy—currently 82.4 years for females and 76.5 for males—and a sustained decline in the fertility rate that mirrors trends in developed countries (2). The increase in the proportion of elderly people has been accompanied by a rise in the number of people with dementia to around 200,000 in 2020, a figure expected to surpass 500,000 by 2050 (3). This figure is especially concerning for a country with almost 20 million people requiring urgent attention from the public and private sectors.

In 2015, the Chilean government launched the first National Plan for Dementia, upgrading the disease to a national priority (4). The biopsychosocial model proposed comprised actions in the health sector and in other areas, including the promotion of dementia-friendly communities (DFC). However, there are no clear definitions or guidelines for the implementation of these communities in the Chilean context. This perspective article considers housing as the primary component for the development of healthy communities and neighbourhoods, focusing on the urban aspects of social housing policy, such as placement, networks, affordability, and the relationship between the subsidy structure and adequate housing provision for people living with dementia in Chile.

## Caring for People Living With Dementia in Chile

In Chile, as in most Latin American countries, people living with dementia, along with their caregivers, are struggling silently against inadequate support and the economic cost of treating the disease (5). Patients and families feel abandoned by a broken health and social care system, relying on unspecialised support from relatives and neighbours. Some 97% of people with dementia live in a family member's home, and in over 70% of cases are cared for by a female caregiver, generally daughters or spouses (6). Informal care delivered by family members who are inactive in the labour market increases indirect costs in lower socio-economic status groups (7). This scenario contrasts with the situation in high-income countries, where up to 50% of people living with dementia reside in care homes (8).

In this context and following recommendations by the World Health Organization (WHO) and Alzheimer's Disease International (9), the Chilean government, headed by Michelle Bachelet, delivered the first Plan for Dementia in 2015 (4). This national strategy was developed by an intersectoral work group comprising—in recognition of the wide range of medical and social assistance concerned—stakeholders from civil society, academics, clinicians, and decision-makers involved in dementia care. The result was the enactment of the National Plan for Dementia in 2017. This policy guideline encompasses actions for dementia in the health sector and in other areas, including the promotion of DFC and age-friendly cities in the long term (10).

The Chilean plan's DFC approach prioritises action to create awareness, reduce stigma and foster dementia-friendly environments. The strategy considers multi-sectoral collaboration for the design and construction of new infrastructure and public spaces to provide physical environments for social participation by people living with dementia. It is expected that the Ministry of Health, along with the Ministry of Housing and City Planning and the National Service for the Older People will coordinate multidisciplinary work with universities, scientific societies, municipalities, media and civil society towards creation of a dementia-friendly environment. However, no details have been provided about the initiative and there is no specific budget or investment for this specific goal.

## Framing Dementia-Friendly Communities in Chile

The concept of DFC has recently been embraced by dementia researchers and policymakers to ensure a better life for people with dementia and their families. However, there are a variety of perspectives on the concept, indicating constant reconsideration of the issue (11). For instance, the WHO defines DFC as an approach that normalises dementia in society. This is aligned with Alzheimer's Disease International, which emphasises empowerment, self-confidence and participation in meaningful activities as the defining attributes of DFC (12). Nevertheless, local initiatives in the UK and Australia have defined DFC based on the responsiveness of the physical and social environment of a person with dementia to preserve access to local facilities and their social networks (13, 14). As there is no single model for developing DFC nor a template for expected outcomes, the definition must be interpreted in the social and physical context in which these communities are developed.

Although the creation of dementia-friendly environments is beginning to gain interest in Latin America and the Caribbean, most countries that include efforts to develop DFC in their national strategies have prioritised raising awareness of dementia over interventions in the physical environment. For example, educational, social and awareness events have taken place in Argentina, Brazil and Costa Rica, led mainly by local Alzheimer's associations and with far less government involvement (15). Increased awareness and understanding of dementia remains a major concern in the region and one of the outcomes of any DFC (12). However, the ageing process that many low- and middle-income countries are already experiencing will reveal the need for a more comprehensive approach in which both the social and physical environment are seen as essential to the formation of ageing- and dementia-friendly societies.

The definitions of community as applied to the development of DFC remain diverse and may constitute a space, a social environment or even an organisation or virtual community (11, 16). In the case of Chile, where most patients with dementia live in the family home, neighbourhoods play a key role in promoting well-being and quality of life for people living with dementia and their families. Although the impact of the neighbourhood has been largely ignored by the biomedical model, epidemiological evidence supports its role as an additional determinant of health that modulates potential risk factors at the individual level. Neighbourhoods that support active ageing may reduce dementia risk factors, while unfavourable environmental features such as low green space availability and poor access to local services might have the opposite effect (17–19).

In this perspective, we recognise that the dementia-friendly community approach requires not only a social and educational intervention but also urban and environmental adaptation. This is mainly because a community is based on a space—a territory in which there are socio-spatial relations that maintain bonded networking. Temporality is considered a key element here because it allows neighbours to identify and include themselves within collective history (20). This means that members of the community are aware of who is part of it and broadly comprehend the situation in which participants are related to them. These bonds, based on time and location, are especially important in low-income contexts, where survival relies on the social networking that sustains everyday life through practises of collaboration and solidarity. Women have a key role here, as the structural division of labour pushes them to undertake work involved in social reproduction (21).

In this sense, a community represents a safe space that can offer diverse networks rich enough to facilitate support when needed. Regarding dementia, we consider at least three urban features to be important and require special attention in relation to the development of DFC. The first is secure housing tenancy. It is generally agreed that insecurities in housing tenancy could drive both mental and physical health issues for householders (22, 23). The second feature is provision of public care infrastructure that is well-distributed across cities. Here, specialised centres could help in providing not only medical but also educational support to make neighbours aware of the needs of people with dementia (11). The third feature is the adaptation to these special requirements of not only housing units but also the urban environment as a whole in order to render neighbourhoods inhabitable by and bearable for those who live with dementia, especially in terms of safe mobility (24, 25) and urban equipment.

## The Impact of Displacement and Forced Eviction on People Living With Dementia

Given that spatial injustice inflicting disproportionate damage on vulnerable population (26), older people and especially those suffering from physical and mental disabilities should be among the groups to receive priority attention from urban and healthcare policies. “Ageing in place,” or the ability to remain rooted in one's own home and community rather than in a residential home, regardless of age and income, is preferred by most older people (27).

For older people with dementia and their families, the concept of place goes far beyond houses and encompasses a community based on a neighbourhood that can be mobilised to improve adaptation and self-management. Yet the term is elusive in public policy and there is a tendency to treat place simply as a home instead of a socially interconnected system, despite growing evidence for the contribution of integrated neighbourhoods and communities to well-being in old age (28). This social interaction is crucial to allowing older people to maximise their well-being despite chronic medical conditions and thus to establishing a new definition of health from a social perspective (29).

The aspiration of ageing in a familiar environment is under threat from evictions—a global phenomenon related to gentrification occurring in both developing and developed countries (30) and affecting mainly poor and vulnerable communities (31, 32). The negative consequences of displacement for people with dementia can be predicted based on evidence that reveals the impact of an abrupt change in the usual physical and social environment. For example, the transition to a nursing home represents a challenging experience for patients, associated with loss of home, neighbourhoods and daily contact with close family members, and may result in poorer mental and physical health (33, 34). Similarly, changes in the usual environment of older people with dementia, such as prolonged hospitalisation, increase the risk of delirium, an acute and life-threatening attention and cognitive disorder that leads to loss of independence and increased morbidity and mortality (35, 36). As such, displacement of older people with dementia to an unfamiliar neighbourhood is likely to harm well-being and behaviour.

## Barriers to DFC in Relation to the Chilean Housing Crisis

Chilean legislation does not consider housing as a right. Instead, it is covered by the right to property, which views housing as a market-tradable commodity (37). In other words, access to housing is dependent on the financial resources of each individual or family. Further, land was deregulated in 1979, giving total control of urban development to the market (38). Together, this has led to a constant rise in prices and speculation in terms of housing stock and its construction (39, 40), increasing social inequality (41), segregation (42, 43) and exclusion (44, 45).

The state does not have the power to directly manage the housing deficit, which has recently reached 497,560 housing units (46). The role of the state is limited to the delivery of subsidies through various programmes focused on demand. As a result, social housing remains excluded from more established, central urban areas due to land prices (47). As a result, people living in poverty have been constantly displaced from their neighbourhoods to areas lacking in urban infrastructure, services, and amenities.

The subsidy programmes consist of quarterly contests during which funds are allocated depending on the vulnerability score of applicants (48). Level of vulnerability is measured parametrically using a tool called the Household Social Registry (HSR, *Registro Social de Hogares*). The instrument classifies individuals by percentage based on the situation of their family group, addressing factors such as their educational level, housing condition, total income, physical and mental health, and access to social security. Importantly, the HSR awards more credits to people who care for others, especially elderly people who are completely dependent upon them (49). This means that informal and unpaid caregivers to the elderly are more likely to be categorised within the most vulnerable socio-economic section of society, thus increasing their likelihood of receiving public funds over people who care for children and teenagers.

With this in mind, it is concerning that more than 1.3 million people in Chile declare themselves to work as unpaid carers of a relative. Some 97% of these caregivers are women (50), and their situation has a considerable negative impact on their educational and career trajectories. As such, there is a strong connection between this unpaid female labour and the percentage of women who apply for and receive housing subsidies, a figure which today stands at 81% (51). It could be said there is some recognition on the part of the HSR of the vulnerability of caregivers engaged in unpaid labour. However, this is not to say that subsidies necessarily cover the special needs of housing programme beneficiaries, especially in terms of their disabilities and/or mental health conditions.

As mentioned previously, one aspect used to measure vulnerability is the health situation of the individual and their capacity for independence from the householder. The survey offers six selectable options concerning health condition, plus five relating to activities that could be completed by the individual, for example, whether they are able to be alone in public spaces. However, it not possible to relate answers with a particular diagnosis beyond evident conditions such as blindness or physical impairments. For example, the options presented in the survey fail to differentiate between mental issues and psychiatric problems, and people often struggle to answer the question. As such, data is not collected specifically about people living with dementia, their degree of vulnerability, or their socio-economic situation. Indeed, dementia could be confused with other conditions, obscuring valuable data needed to effect material improvements to the environment. We interpret this as a lack of political will to address and improve the situation, a view reinforced by the meagre 0.06% of central government budget that is assigned to elderly programmes (52), among which the amount available to dementia programmes is unclear and unstable.

As we have observed through our ethnographic experience of working with women's housing committees, this obscuring of people's health conditions has at least three potential side effects for people living with dementia. Generated by a social housing policy that we consider to constitute a threat to dementia sufferers, these side effects are (a) the double vulnerabilisation of people, whereby applicants attempt to achieve the figures needed to receive subsidies, thus exposing family members to risky conditions in the process; (b) the displacement of caregivers—with or without their respective dementia sufferer—from their original neighbourhoods to underdeveloped satellite settlements; and (c) the tendency for overcrowding through the construction of informal and dangerous house extensions as people struggle to maintain their network of care and avoid eviction by the housing market or state subsidies.

The first of these risks is directly related to the healthcare system. The HSR awards higher scores to those whose healthcare coverage is provided by the public system (49). This creates a dilemma for families who cannot afford to buy or rent a home but whose members are affiliated with the private health system, which is far more expensive and effective than the public system. It is common for families in this situation to opt to expose themselves to poorer healthcare coverage, thus doubling their initial condition of vulnerability. This state of affairs can persist throughout the housing application process, which frequently lasts more than 5 years.

The second risk stems from the fact that, as indicated earlier, an important condition for creating dementia-friendly communities concerns allowing people to reside in the place with which they are most familiar. Subsidy beneficiaries tend to be displaced from their original neighbourhoods to urban peripheries (53), causing a two-fold problem. First, if the caregiver is displaced, their continued care work could be rendered unfeasible by long commutes. The effect of this on people living with dementia could be substantial, as extra effort would be involved in comprehending an unexpectedly changed relationship with the caregiver. A second problem would arise if both sufferer and caregiver are displaced, resulting in the loss of the community network which sustains them.

This situation requires additional psychological effort on the part of the dementia sufferer, which, as mentioned above, would also affect their quality of life, as they must come to understand and navigate an entirely new environment. In addition, urban peripheries in Chile lack social services and urban amenities, exposing people with dementia to loss of access to healthcare facilities and stress generated by long journeys. Furthermore, it is recognised that caregivers tend to receive frequent support from various people involved in their care network, all of whom live in the same neighbourhood. Displacement would mean loss of this essential support and potential psychological, social and economic impacts as displaced people find themselves paying for all of the assistance and services previously provided by neighbours and relatives.

The third risk is the phenomenon of overcrowding, related to the housing affordability crisis and job insecurity (54) triggered by the transformation of spatial design in both residential and urban areas. Here, kinship is a crucial factor, as householders receive relatives into their homes in order to save them from homelessness or displacement. However, this involves informal deconstruction/reconstruction of housing spaces in order to adapt to growing occupant numbers. The process has several detrimental effects on the quality of life of inhabitants, mostly associated with precarious and often extremely risky adaptations to homes (55). Healthcare and sanitary risks are high, and the COVID-19 pandemic has exacerbated the situation due to the challenges of maintaining physical and social distance (56). This has a direct impact on elderly people who find themselves in disrupted environments and new undefined social relations of co-dependency—conditions that also tend to increase instances of domestic violence (57).

## Conclusion and Recommendations

The rapid ageing of Chile's population over the last few decades has emphasised the need for protection of the elderly and older people living with dementia. The dynamics of dementia care in Chile depend on the socio-spatial connections established during the sufferer's lifetime, and ageing in place is thus a cornerstone for the implementation of dementia-friendly communities. Nevertheless, housing shortages driven by constant price rises associated with speculation and the subsidiary housing model expose poor and vulnerable communities to the negative consequences of displacement.

From a public health perspective, we recommend a review of current housing and land policy in view of the considerable impact of urban areas on the physical and mental well-being and care of people, especially those with disabilities. The recently initiated constitutional process provides key political momentum for this, and it is hoped that improvements will be made to the development of healthcare and urban spaces, moving from the subsidiary model to the politics of distribution and recognition.

## Data Availability Statement

The original contributions presented in the study are included in the article/supplementary material, further inquiries can be directed to the corresponding author/s.

## Author Contributions

DJ and FC-C: article concept and design, literature research, drafting of the manuscript, critical revision, and final approval of the manuscript. Both authors contributed to the article and approved the submitted version.

## ACKNOWLEDGEMENTS

We thank the members of the *Kintun* programme for families with dementia and the EAGIS team from the *Movimiento de Pobladoras y Pobladores en Lucha* for their valuable insights.

## Conflict of Interest

The authors declare that the research was conducted in the absence of any commercial or financial relationships that could be construed as a potential conflict of interest.

## Publisher's Note

All claims expressed in this article are solely those of the authors and do not necessarily represent those of their affiliated organizations, or those of the publisher, the editors and the reviewers. Any product that may be evaluated in this article, or claim that may be made by its manufacturer, is not guaranteed or endorsed by the publisher.
